# Characterization of Pannexin1, Connexin32, and Connexin43 in Spotted Sea Bass (*Lateolabrax maculatus*): They Are Important Neuro-Related Immune Response Genes Involved in Inflammation-Induced ATP Release

**DOI:** 10.3389/fimmu.2022.870679

**Published:** 2022-04-19

**Authors:** Zhaosheng Sun, Chong Xu, Yuxi Chen, Danjie Liu, Ping Wu, Qian Gao

**Affiliations:** ^1^Key Laboratory of Exploration and Utilization of Aquatic Genetic Resources, Ministry of Education, Shanghai Ocean University, Shanghai, China; ^2^International Research Center for Marine Biosciences at Shanghai Ocean University, Ministry of Science and Technology, Shanghai, China; ^3^National Demonstration Center for Experimental Fisheries Science Education, Shanghai Ocean University, Shanghai, China; ^4^College of Life Science and Technology, Huazhong University of Science and Technology, Wuhan, China

**Keywords:** pannexin1, connexin32, connexin43, innate immunity, ATP release, *Lateolabrax maculatus*

## Abstract

Many immunological diseases can be treated by regulating neurobehavior, in which extracellular ATP is a vital member of endogenous danger-associated molecular pattern signaling molecule that plays a crucial part in innate neuro-related immunity. It is actively released through pannexin (Panx) and connexin (Cx) hemichannels from activated or stressed cells during inflammation, injury, or apoptosis. In addition to participating in ATP release, Panxs and Cxs also have crucial immune functions. In this study, pannexin1, three connexin32 isoforms and connexin43 were identified and characterized in spotted sea bass (*Lateolabrax maculatus*), which were named *Lm*Panx1, *Lm*Cx32.2, *Lm*Cx32.3, *Lm*Cx32.7, and *Lm*Cx43. Their similar topological structures were discovered by sequence analysis: a relatively unconserved C-terminal region and four highly conserved transmembrane (TM) domains, and so on. Each extracellular (ECL) region of Panx1 has two conserved cysteine residues. Unlike Panx1, each ECL region of Cx32 and Cx43 contains three conserved cysteine residues, forming two conserved motifs: CX_6_CX_3_C motif in ECL1 and CX_4_CX_5_C motif in ECL2. Furthermore, Panx1 and Cx43 share similar genomic organization and synteny with their counterparts in selected vertebrates. Cx32 and CX43 were located in the same locus in fish, but diverged into two loci from amphibian. Moreover, despite varying expression levels, the identified genes were constitutively expressed in all examined tissues. All genes were upregulated by PAMP [lipopolysaccharide and poly(I:C)] stimulation or bacterial infection *in vivo* and *in vitro*, but they were downregulated in the brain at 6 or 12 h after stimulation. Especially, the three *Lm*Cx32 isoforms and *Lm*Cx43 were upregulated by ATP stimulation in primary head kidney leukocytes; however, downregulation of *Lm*Cx32.3 and *Lm*Cx43 expression were noted at 12 h. Conversely, ATP treatment inhibited the expression of *Lm*Panx1. Importantly, we showed that the spotted sea bass Panx1, Cx43, and Cx32 were localized on the cellular membrane and involved in inflammation-induced ATP release. Taken together, our results demonstrated that Panx1, Cx32, and Cx43 are important neuro-related immune response genes involved in inflammation-induced ATP release.

## Introduction

Regulating neurobehavior shows great promise for therapeutic application in a variety of immunological diseases and clinical conditions (1). For examples, sepsis can be treated by electrical stimulation of the vagus nerve (1), and electrical stimulation can promote the flow of calcium ions into nerve cells and the synthesis and release of ATP (2, 3). As a considerable member of endogenous danger-associated molecular pattern signaling molecule, extracellular ATP plays vital roles in natural immunity (4). In mammals, extracellular ATP is actively released from activated or stressed cells during inflammation, injury or apoptosis (5, 6). What’s more, extensive immune response and inflammation can also be caused by extracellular ATP (6). In particular, extracellular ATP as a primary afferent neurotransmitter participated in the process of neuro-immune interactions (7). In addition, extracellular ATP involved in the process of pro-inflammatory cytokines (IL-1β, caspases, IFN and Mx, etc.) release (8–11) and the activation of the NLRP3/NLRC3 inflammasome (12–15). Therefore, extracellular ATP was considered as an effective signaling molecule to activate the natural immune responses (16). Apparently, it is extraordinarily important to study the molecular determinants for inflammation-induced ATP release.

Connexins (Cxs) are the main members of gap junctions, which control several second messengers to diffuse between adjacent cells (17). In addition, some Cx members can form hemichannels involved in extracellular ATP release (18). Since the first Cx gene was cloned, 21 isoforms of Cxs have been identified in mammals, such as Cx26, Cx32, and Cx43 (19). They have been identified as an important component of the cell homeostasis, differentiation, inflammation and natural immune responses (20, 21). About the Cx family, Cx32 and Cx43 play a role in a variety of immune cells and participate in the release of ATP (22, 23). Consequently, Cx32 and Cx43 have been proven to play vital immune roles (24, 25).

In addition to Cxs, pannexin (Panx) hemichannels were also involved in ATP release in mammals (26). Panxs are membrane channels glycoproteins with a similar topological structure to Cxs, including four transmembrane (TM) domains, one intracellular loop, two extracellular loops, and both the N- and C- terminal regions being intracellular (27). The Panx protein family includes three members, Panx1, Panx2, and Panx3. Like Cx32 and Cx43, Panx1 is expressed in several kinds of immune cells and plays very crucial roles in physiological and pathological processes in mammals, especially in inflammasome activation (28), cytokine release (28), and T cell activation (29).

Even though the functions of Cxs and Panxs in ATP release and immune responses have been extensively studied in mammals, their effects remain not clear in fish. Several recent studies have shown that Panx1 (4), Cx32 (21), and Cx43 (30) are important immune response genes and play an essential role in inflammation-induced ATP release in Japanese flounder (*Paralichthys olivaceus*). These results first showed that the Panx1, Cx32, and Cx43 genes are involved in innate immunity in fish. Panx1 is also an important immune response gene involved in bacterial infection-induced ATP release in tilapia (*Oreochromis niloticus*) (31). Besides, Cx and Panx genes were constitutively expressed in all the tissues and have been shown to respond to the bacterial infection in turbot (*Scophthalmus maximus* L.) (32). Taken together, the evidence that Panxs and Cxs were involved in ATP release and innate immune responses in fish remains immensely limited.

As a vital commercial fishes, spotted sea bass (*Lateolabrax maculatus*) widely farmed in East Asia because of their high nutritional value and adaptation to various salinity waters (seawater, brackish water and freshwater, etc.) (33, 34). Further study on the innate immunity of *L. maculatus* will help to understand its immune mechanisms. In this study, Panx1, three Cx32 isoforms and Cx43 were identified and characterized in *L. maculatus*, which were named *Lm*Panx1, *Lm*Cx32.2, *Lm*Cx32.3, *Lm*Cx32.7, and *Lm*Cx43, and gene expression was analyzed by PAMP [lipopolysaccharide (LPS) and poly(I:C)] stimulation or bacterial infection *in vivo* and *in vitro* using real-time quantitative polymerase chain reaction (qPCR). Importantly, we determined their subcellular localization and explored their function in inflammation-induced ATP release. Our findings will contribute to further understanding of the innate immune response mediated by extracellular ATP in fish and of neuroimmunity in vertebrates.

## Materials and Methods

### Experimental Fish

*L. maculatus* (100 ± 10 g) were farmed in a freshwater fish aquaculture system at 26°C ± 2°C for more than 2 weeks prior to experiments. Fish were sourced from a fish freshwater farm in Hangzhou city, Zhejiang province, China.

### Cloning of Panx1, Cx32, and Cx43 From Spotted Sea Bass

Total RNA was extracted, and partial gene sequences were obtained as described previously (34). The full-length cDNA sequences of *Lm*Panx1, *Lm*Cx32.2, *Lm*Cx32.3, *Lm*Cx32.7, and *Lm*Cx43 were then cloned and verified by our previous methods (35). All primers are summarized in **Table 1**.

**Table 1:** Primers used for cloning and real-time PCR.

Primers	Sequence (5′ to 3′)	Application
*Lm*Panx1-F1	CTTGGCGGGAGCAGTGGTTG	Sequence validation
*Lm*Panx1-R1	AGAGTCCGTGGCATTCGTTTT	Sequence validation
*Lm*Panx1-3F1	CTCGTCAACTTGGTCCTGTTCATT	3′-RACE
*Lm*Panx1-3F2	ACGTCAGCGAACTAAAGTCCTACAA	3′-RACE
*Lm*Panx1-5R1	TCACTGCCACCAGCAACAAAA	5′-RACE
*Lm*Panx1-5R2	CTCTGTTCGGATGTTGCGGTAT	5′-RACE
*Lm*Panx1-F2	CCGCCATGAAACTTTAACAGACA	Verify the CDS
*Lm*Panx1-R2	AAATAAATCTGCTCCTCCTTCTTCC	Verify the CDS
*Lm*Cx32.2-F1	AGTGGGGTTTTCTGTCCTCTCTC	Sequence validation
*Lm*Cx32.2-R1	GCTGTGCTGTGACTGGCATCAT	Sequence validation
*Lm*Cx32.2-3F1	CTGTACGGGTTTGTCATGGACC	3′-RACE
*Lm*Cx32.2-3F2	TTCTACCTGGCGTGTTCTCGC	3′-RACE
*Lm*Cx32.2-5R1	TCAGAGTCGGTGTTGAGACAAAGAT	5′-RACE
*Lm*Cx32.2-5R2	TGGGAAGGCATGGTCATAGCAG	5′-RACE
*Lm*Cx32.2-F2	ATTCAGCCCCACAGCAGGTGA	Verify the CDS
*Lm*Cx32.2-R2	TCAAAGAAGAGCTTGCAGCACTAAA	Verify the CDS
*Lm*Cx32.3-F1	ATGGGAGACTTTGGTTTTCTGTCA	Sequence validation
*Lm*Cx32.3-R1	ACCACTCAGCAGTTGTTTCTCCTC	Sequence validation
*Lm*Cx32.3-3F1	CCTGCTCCAAGAAGCCCTGT	3′-RACE
*Lm*Cx32.3-3F2	GATTTGTACCAGGGTCAGATGCG	3′-RACE
*Lm*Cx32.3-5R1	CCCTTGATCGTCACCTTTCCCT	5′-RACE
*Lm*Cx32.3-5R2	CCAGAAGCGAATGTGCGAGAT	5′-RACE
*Lm*Cx32.3-F2	ATCTCTCCCAGCCAGAGACAGTCC	Verify the CDS
*Lm*Cx32.3-R2	CACGCTTTCCATTATGAGATTTCC	Verify the CDS
*Lm*Cx32.7-F1	GGGCGATGAGCAATCTGACTTT	Sequence validation
*Lm*Cx32.7-R1	CCATGTTGTTGTTCTCAGGCGA	Sequence validation
*Lm*Cx32.7-3F1	TCCGTCTCCCTCGTCCTCAGT	3′-RACE
*Lm*Cx32.7-3F2	ATGGCGAGGAGGCAGGACTA	3′-RACE
*Lm*Cx32.7-5R1	GCCACCAGCATGAACCAGATG	5′-RACE
*Lm*Cx32.7-5R2	CTGGAGGGTGAAACCGTAAAGT	5′-RACE
*Lm*Cx32.7-F2	AAGCAGGACAACTGGCGACTGAA	Verify the CDS
*Lm*Cx32.7-R2	CACACCGTTTAACTTCCCCAACG	Verify the CDS
*Lm*Cx43-F1	CTGGGTCGTCTACTGGACAAGG	Sequence validation
*Lm*Cx43-R1	CTTATGCTCGTGGGTATCATCG	Sequence validation
*Lm*Cx43-3F1	TGGTGTCCCTGCTGCTCAAC	3′-RACE
*Lm*Cx43-3F2	CTGTCCGCTGCTAAGTACGCT	3′-RACE
*Lm*Cx43-5R1	CGTGCTCCTCAATGCCATACTTT	5′-RACE
*Lm*Cx43-5R2	TGGGTGTTACATTTGAAGGCAGA	5′-RACE
*Lm*Cx43-F2	CGGTCCCAAACTTGGATTTC	Verify the CDS
*Lm*Cx43-R2	GACAACAGTGATTGAGGTTAGCC	Verify the CDS
*Lm*Panx1-qF	CTGAGGAGACGAGGTCATTGC	Real-time PCR
*Lm*Panx1-qR	GCAAGGGAGTGAGCTCTTTCATC	Real-time PCR
*Lm*Cx32.2-qF	AAGGAGACCTGCTGGGAAACTAC	Real-time PCR
*Lm*Cx32.2-qR	CGAGAACACGCCAGGTAGAAG	Real-time PCR
*Lm*Cx32.3-qF	GATTTGTACCAGGGTCAGATGCG	Real-time PCR
*Lm*Cx32.3-qR	CCATCCAGGCTTCCACCAATAC	Real-time PCR
*Lm*Cx32.7-qF	CCCTCGTCCTCAGTCTGGTTG	Real-time PCR
*Lm*Cx32.7-qR	TGTTCTCAGGCGATACGTTCTTG	Real-time PCR
*Lm*Cx43-qF	ACCAATGTCCCCTCCAGGCTAC	Real-time PCR
*Lm*Cx43-qR	TTATGCTCGTGGGTATCATCGG	Real-time PCR
*Lm*EF1α-qF	ATCTCTGGATGGCACGGAGA	Real-time PCR
*Lm*EF1α-qR	CAGTGTGGTTCCGCTAGCAT	Real-time PCR

### Sequence Analysis of *Lm*Panx1, *Lm*Cx32, and *Lm*Cx43

Programs on the NCBI website (https://www.ncbi.nlm.nih.gov/) and Expasy website (http://www.expasy.org) were used to analyze nucleotide and protein sequences. Phylogenetic trees and multiple sequence alignment of Panx1, Cx32, and Cx43 were analyzed using the ClustalW, GeneDoc, and MEGA 5.1 program, according to the method described previously (35). The Ensembl and NCBI genome databases were analyzed to infer the genomic organization and syntenic relationships.

### Tissue Expression of *Lm*Panx1, *Lm*Cx32, and *Lm*Cx43

Eight tissue samples (head kidney, spleen, gill, intestine, brain, liver, skin, and muscle) were obtained from healthy *L. maculatus* and used for total RNA extraction by TRIzol reagent. Total RNA was then reversed to cDNA for qPCR according to the method described previously (34). All primers are summarized in **Table 1**.

### Expression of *Lm*Panx1, *Lm*Cx32, and *Lm*Cx43 in Spotted Sea Bass to the PAMP or *Edwardsiella tarda* Challenge

*L. maculatus* were intraperitoneal (i.p.) injected with 500 µL *Edwardsiella tarda* [1 × 10^5^ colony-forming units (CFU)/mL], LPS (1 mg/mL), poly(I:C) (1 mg/mL), or phosphate-buffered saline (PBS) (control) for the challenge experiments by our previous methods (34). Each condition was done in quadruplicate. Next, according to the method described previously, tissue samples were obtained at 6, 12, 24, and 48 h after injection, and total RNA was then reversed to cDNA for qPCR. The *E. tarda* were prepared as previously described (34, 36). LPS and poly(I:C) were purchased from Sigma–Aldrich (USA).

### Expression of *Lm*Panx1, *Lm*Cx32, and *Lm*Cx43 in Primary Head Kidney Leukocytes

Primary head kidney leukocytes were isolated by using a discontinuous Percoll gradient as previously described (37). The leukocytes cultured in a six-well plate (Corning, United States) with DMEM-F12 complete medium [DMEM-F12 with 10% fetal bovine serum and 1% Pen/Strep (penicillin/streptomycin)] in a CO_2_ incubator at 28°C. The leukocytes (1 × 10^7^/well) were treated with LPS (100 µg/mL), poly(I:C) (50 µg/mL), and ATP (100 µM or 1 mM), respectively. Each condition was done in quadruplicate. Cell samples were collected at 6, 12, 24, and 48 h after stimulation, and total RNA was then reversed to cDNA for qPCR. ATP was purchased from Sigma–Aldrich, and all cell culture reagents were purchased from Gibco (USA).

### Subcellular Localization

The pEGFP-N1 expression plasmid containing the coding sequence (CDS) of *Lm*Panx1, *Lm*Cx32s, or *Lm*Cx43 were constructed, that is, pEGFP-N1-*Lm*Panx1, pEGFP-N1-*Lm*Cx32.2, pEGFP-N1-*Lm*Cx32.3, pEGFP-N1-*Lm*Cx32.7, and pEGFP-N1-*Lm*Cx43. We then transfected the recombinant pEGFP-N1 plasmids into HEK293 T cells by the same methods as before (35). The transfected cells were cultured at 24 h and treated by the method described previously (38). Next, cells were stained with DAPI (Solarbio, China) and observed using a laser confocal microscope (Leica TCS SP8, Germany). All cell culture reagents were purchased from Gibco.

### Extracellular ATP Measurement

To examine the LPS-induced extracellular ATP release in primary head kidney leukocytes of *L. maculatus*, the leukocytes (1 × 10^5^/well) were cultured in a 24-well plate (Corning, United States) and stimulated with LPS (100 µg/mL) or PBS. The supernatants were collected at 15 and 30 min after stimulation and used to measure extracellular ATP levels. Each condition was done in quadruplicate. The ATP release level was measured with Enhanced ATP Assay Kit (Beyotime, China) in LumiPro (YPHBIO, China). All cell culture reagents were purchased from Gibco.

To explore the role of *Lm*Panx1, *Lm*Cx32, and *Lm*Cx43 in LPS-induced extracellular ATP release, the pcDNA3.1 expression plasmid containing the CDS of *Lm*Panx1, *Lm*Cx32s, or *Lm*Cx43 was constructed, that is, pcDNA3.1-*Lm*Panx1, pcDNA3.1-*Lm*Cx32.2, pcDNA3.1-*Lm*Cx32.3, pcDNA3.1-*Lm*Cx32.7, and pcDNA3.1-*Lm*Cx43. The same number of HEK293 T cells transfected with expression plasmids or empty plasmid (pcDNA3.1) was cultured in a 24-well plate at 24 h, and then were stimulated with LPS (100 µg/mL). Meanwhile, the mock transfected and empty plasmid transfected cells (negative controls) were cultured in another 24-well plate at 24 h, but were not stimulated with LPS. The supernatants were collected at 15 and 30 min after stimulation and used to measure extracellular ATP levels. Each condition was done in quadruplicate. The ATP levels were then measured as described previously.

### Statistical Analysis

The data were processed and statistically analyzed using the IBM SPSS package (SPSS 20.0, SPSS Inc., Chicago, IL, United States). Significant differences (*p* < 0.05 or *p* < 0.01) between experimental groups and control groups were analyzed using analysis of variance as previously described (35).

## Results

### Sequence Identification of *Lm*Panx1, *Lm*Cx32, and *Lm*Cx43

The Panx1, Cx32, and Cx43 sequences in *L. maculatus* were submitted to the GenBank database: OM315303 (*Lm*Panx1), OM315304 (*Lm*Cx32.2), OM315305 (*Lm*Cx32.3), OM315306 (*Lm*Cx32.7), and OM315307 (*Lm*Cx43).

**Figure 1:**
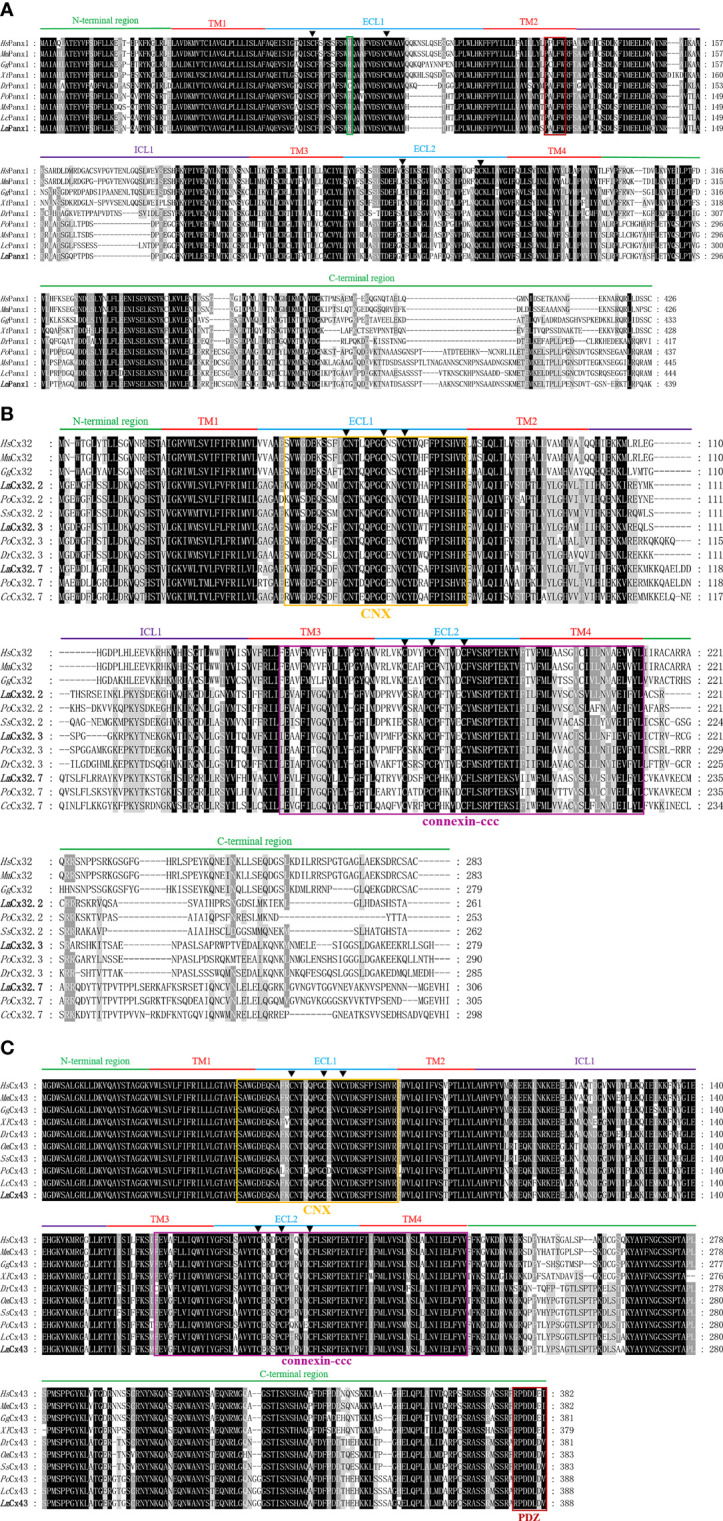
Multiple sequence alignment analysis of Panx1 **(A)**, Cx32 **(B)**, and Cx43 **(C)**. The N-terminal region, four TM domains (TM1-4), the intracellular loop (ICL1), the extracellular loops (ECL1-2), and the C-terminal region of *Lm*Panx1, *Lm*Cx32, and *Lm*Cx43, are marked above the alignment. Symbol (▲) indicates the conserved cysteine residues. The K or R residue in position 75 and the classic innexin-specific P-X-X-X-W motif are boxed in green and red, respectively. In addition, the CNX domain, connexin-ccc domain, and PDZ domain are boxed in yellow, purple, and red, respectively. The *Lm*Panx1, *Lm*Cx32, and *Lm*Cx43 are shown in bold. The accession numbers of sequences are shown in **Figure 2**. *Hs*, *Homo sapiens*; *Mm*, *Mus musculus*; *Gg*, *Gallus gallus*; *Xt*, *Xenopus tropicalis*; *Dr*, *Danio rerio*; *Po*, *Paralichthys olivaceus*; *Ms*, *Morone saxatilis*; *Lc*, *Larimichthys crocea*; *Lm*, *Lateolabrax maculatus*; *Ss*, *Salmo salar*; *Cc*, *Cyprinus carpio*; *Om*, *Oncorhynchus mykiss*.

It can be observed from **Supplement Figure 1** that the total length cDNA of *Lm*Panx1 has been cloned, which contains 1,959 bp including a 161-bp 5′ untranslated region (UTR), an open reading frame (ORF) with 1,320 bp encoding 439 amino acids (aa), and a 478-bp 3′-UTR. Moreover, there is a polyadenylation signal (ATTAAA) at the 3′-UTR of the sequence (**Supplement Figure 1**). Multiple sequence alignment revealed that *Lm*Panx1 retains four highly conserved TM domains; meanwhile, it can be observed that TM2 has a typically innexin-specific P-X-X-X-W motif (4) (**Figure 1A**). Each of the extracellular (ECL) regions has two conserved cysteine residues distinctly, and all species except zebrafish contain a charged K or R residue relative to position 75 (**Figure 1A**), which was deemed to be involved in ATP-mediated channel regulation (39).

For the whole cDNA of *Lm*Cx32.2, it is 1,496 bp, with a 71-bp 5′-UTR, an ORF contains 786 and a 639-bp 3′-UTR containing a tail-adding signal (AATAAA) (**Supplement Figure 2A**). The total length of the cloned cDNA sequence of *Lm*Cx32.3 is 1,415 bp, including a 70-bp 5′-UTR, a 840-bp ORF, and a 505-bp 3′-UTR containing a polyadenylation signal (AATAAA) (**Supplement Figure 2B**). The cDNA of *Lm*Cx32.7 was obtained by cloning with 1,357 bp in length, including a 23-bp 5′-UTR, a 921-bp ORF, and a 413-bp 3′-UTR containing a tail-adding signal (AATAAA) (**Supplement Figure 2C**). After multiple sequence alignment, it was found that *Lm*Cx32s contain four conserved TM domains, a connexin homolog (CNX) domain, and a connexin-ccc domain (30). Unlike Panx1, three conserved cysteine residues in each of the ECL region form two conserved motifs, that is, CX_6_CX_3_C motif in ECL1 and CX_4_CX_5_C motif in ECL2 (**Figure 1B**), which play important roles in Cx channel formation (40).

As shown in **Supplement Figure 3**, the full-length cDNA of *Lm*Cx43 is 2,625 bp, with a 1,167-bp ORF encoding a protein of 388 aa. There are 129 bp of 5′-UTR and 1,329 bp of 3′-UTR on either side of the ORF area, and a polyadenylation signal (ATTAAG) at the 3′-UTR. By multiple sequence alignment, the *Lm*Cx43 protein exhibits a high degree of conservation and contains a CNX domain, a connexin-ccc domain, and a PDZ domain. Like Cx32s, three conserved cysteine residues in each of the ECL region form two conserved motifs: CX_6_CX_3_C motif in ECL1 and CX_4_CX_5_C motif in ECL2 (**Figure 1C**).

**Figure 2:**
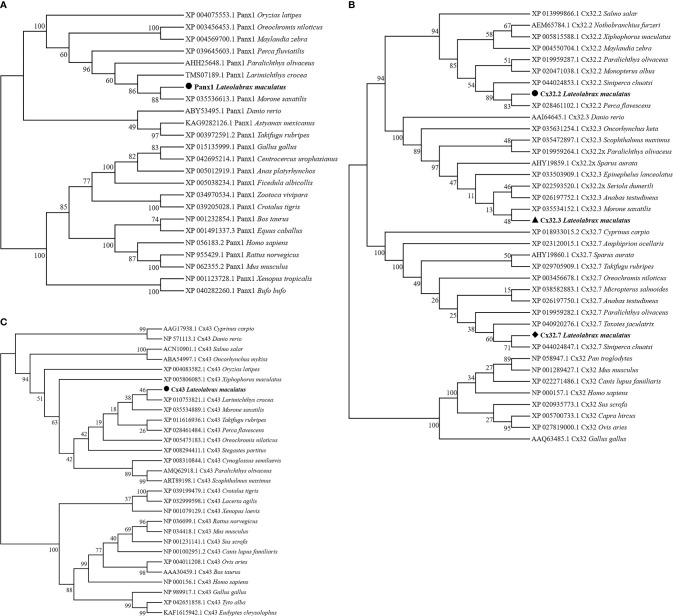
Phylogenetic tree analysis of Panx1 **(A)**, Cx32 **(B)**, and Cx43 **(C)**. Phylogenetic tree was constructed using the NJ method and run for 10,000 replications. The *Lm*Panx1, *Lm*Cx32, and *Lm*Cx43 are shown in bold.

Homologous relationships between Panx1, Cx32, and Cx43 from various animal species were identified by constructing phylogenetic trees, respectively. In **Figure 2A**, *Lm*Panx1 and of *Morone saxatilis* formed one branch (88% bootstrap support) and then were clustered with homologs from other fish species. Corresponding to the clades formed by Cx32.2, Cx32.3, and Cx32.7 homologs from different fish, *Lm*Cx32s were clustered into three distinct branches (**Figure 2B**). In addition, *Lm*Cx43 and of *Larimichthys crocea* formed a clade with 46% of support rate and then were clustered together with other fish homologs into a clade (**Figure 2C**).

### Genomic Organization and Synteny of *Lm*Panx1, *Lm*Cx32, and *Lm*Cx43

**Figure 3:**
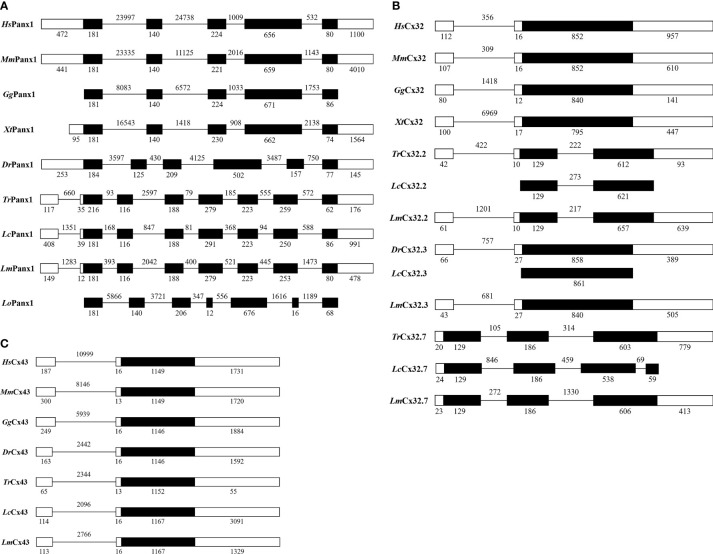
Genomic organization of Panx1 **(A)**, Cx32 **(B)**, and Cx43 **(C)**. Blank and solid boxes indicate UTR and coding exon, respectively. The size (bp) of exons and introns is indicated. Note that the size of exons and introns is disproportionate.

Our study determined the genomic structures of *Lm*Panx1, *Lm*Cx32, and *Lm*Cx43 by comparing their cDNA and genomic sequences (**Figure 3**). There are eight exons and seven introns in *Lm*Panx1 gene; the first intron is located in 5′-UTR, the same organization to that of Fugu, large yellow croaker, and spotted gar Panx1 genes (**Figure 3A**). Besides, the sizes of the first three exons and the last exon of the CDS are comparable with those of Panx1 from selected vertebrates (**Figure 3A**). Similar to Fugu and large yellow croaker, *Lm*Cx32.2, *Lm*Cx32.3, and *Lm*Cx32.7 genes consisted of three, two, and three exons, respectively (**Figure 3B**). Human, mouse, chicken, and frog Cx32 gene had two exons, and the intron is located in 5′-UTR, which is the same as the *Lm*Cx32.3 (**Figure 3B**). The *Lm*Cx43 gene also consisted of two exons, with the same organization as the Cx43 gene in other species (**Figure 3C**).

**Figure 4:**
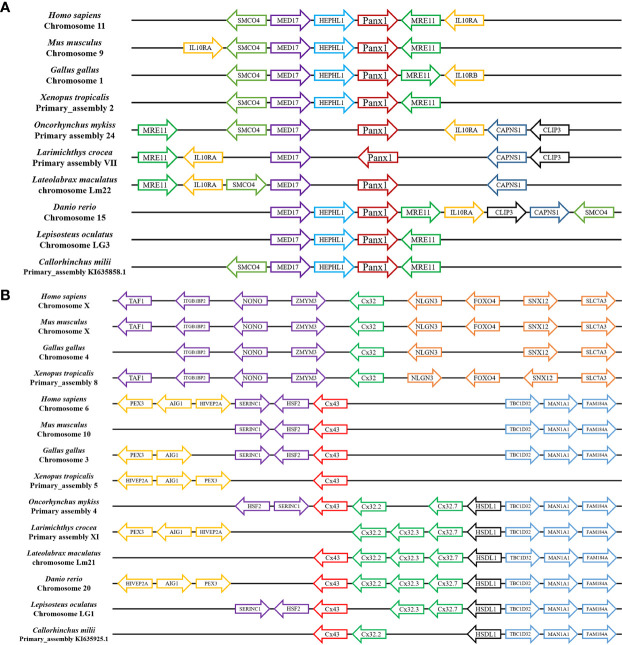
Gene synteny of Panx1 **(A)** and Cx32 and Cx43 **(B)**. The *Lm*Panx1, *Lm*Cx32, and *Lm*Cx43 genome sequence data were obtained from the spotted sea bass genome database (https://www.ncbi.nlm.nih.gov/genome/43909). Synteny information for other vertebrates was retrieved from the Ensembl database (http://www.ensembl.org/index.html). Arrows indicate transcription orientations.

Gene synteny showing that the Panx1 loci have been well conserved during evolution, where the Panx1 linked to MRE11 and MED17, and other genes found in this locus included SMCO4, HEPHL1, IL10RA, CAPNS1, and CLIP3 (**Figure 4A**). The Cx43 and Cx32s were located in the same chromosome in different fish, forming a gene cluster, but Cx43 and Cx32 were located in different chromosomes in human, mouse, chicken, and frog (**Figure 4B**). At the same time, the Cx43 and Cx32s in different fish were located in the same gene locus as the Cx43 gene of the human, mouse, chicken, and frog, which also contains TBC1D32, MAN1A1, FAM184A, HSF2, SERINC1, and so on.

### Expression of *Lm*Panx1, *Lm*Cx32, and *Lm*Cx43 in Tissues

**Figure 5:**
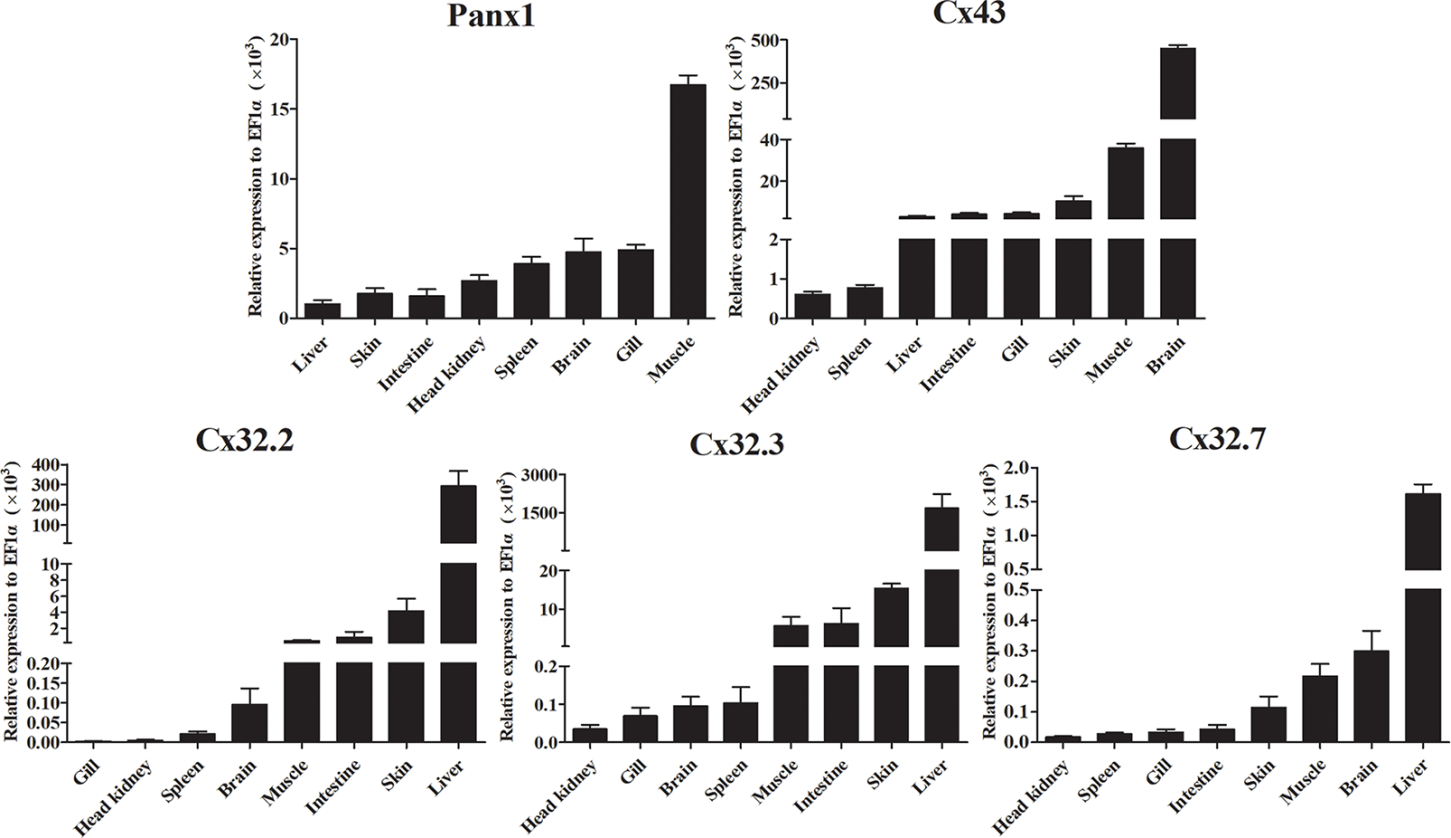
Expression analysis of *Lm*Panx1, *Lm*Cx32, and *Lm*Cx43 in tissues by qPCR. The expression level of each gene was normalized to that of EF1α. Data are presented as mean ± SEM (N = 4).

We analyzed the expression of *Lm*Panx1, *Lm*Cx32, and *Lm*Cx43 in eight tissues, including head kidney, spleen, gill, intestine, brain, liver, skin, and muscle (**Figure 5**). Despite varying expression levels, the identified genes were constitutively expressed in all examined tissues. More specifically, the highest expression levels of *Lm*Panx1, *Lm*Cx32 (*Lm*Cx32.2, *Lm*Cx32.3, and *Lm*Cx32.7), and *Lm*Cx43 were found in muscle, liver, and brain, respectively. In addition, the lowest expression levels of *Lm*Cx32 and *Lm*Cx43 were both found in head kidney. In contrast liver exhibited the lowest expression for *Lm*Panx1, and the moderate expression levels of *Lm*Cx32 and *Lm*Cx43 were found in the intestine, muscle, and skin. Differently, the moderate expression levels of *Lm*Panx1 were found in the brain, gill, and spleen.

### Expression Analysis of *Lm*Panx1, *Lm*Cx32, and *Lm*Cx43 After *In Vivo* Stimulation

**Figure 6:**
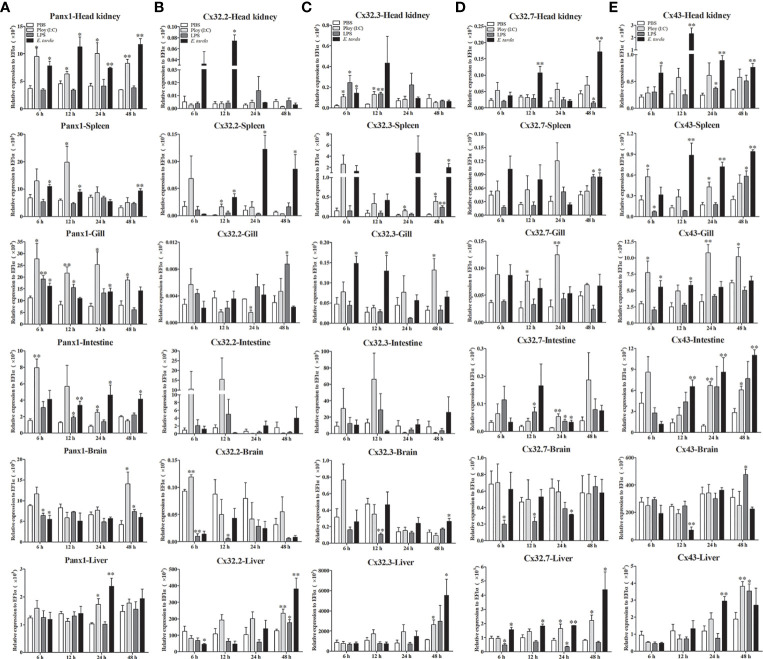
Expression of *Lm*Panx1 **(A)**, *Lm*Cx32.2 **(B)**, *Lm*Cx32.3 **(C)**, *Lm*Cx32.7 **(D)**, and *Lm*Cx43 **(E)** after LPS, poly(I:C), or *Edwardsiella tarda* challenge. Spotted sea bass was i.p. injected with LPS (5 mg/kg body weight), poly(I:C) (5 mg/kg body weight), *E. tarda* (5 × 10^4^ CFU/fish), or PBS (control) The relative expression levels of target genes were normalized to that of EF1α. Data are shown as mean +SEM (N = 4). *p < 0.05, **p < 0.01 are considered significant difference.

The expression patterns of *Lm*Panx1, *Lm*Cx32, and *Lm*Cx43 were analyzed in tissues including the head kidney, spleen, gill, intestine, brain, and liver after PAMP [LPS and poly(I:C)] and *E. tarda* challenge (**Figure 6**). *L. maculatus* were i.p. injected with 500 µL *E. tarda* (1 × 10^5^ CFU/mL), LPS (1 mg/mL), poly(I:C) (1 mg/mL), or PBS for the challenge experiments. Tissue samples were obtained at 6, 12, 24, and 48 h after injection, and total RNA was then reversed to cDNA for qPCR. In head kidney, gill, and intestine, the expression of *Lm*Panx1 was upregulated to the different degrees after three kinds of stimulation. In spleen, the expression of *Lm*Panx1 was upregulated after poly(I:C) (at 12 h) and *E. tarda* (except 24 h) stimulation, but there was no significant change after LPS stimulation; In the brain, *Lm*Panx1 was downregulated at 6 h after LPS and *E. tarda* stimulation, but upregulated at 48 h after LPS and poly(I:C) stimulation; in the liver, *Lm*Panx1 was upregulated only at 24 h after poly(I:C) and *E. tarda* stimulation (**Figure 6A**).

As shown in **Figures 6B–D**, the *Lm*Cx32 isoforms were upregulated in the head kidney, spleen, and liver by the *E. tarda* infection, but downregulated in the brain after LPS stimulation; Furthermore, in the intestine, *Lm*Cx32.7 was upregulated after stimulation (at 24 h), whereas the expression of *Lm*Cx32.2 and *Lm*Cx32.3 remained constant. Moreover, *Lm*Cx32.2 was upregulated in the spleen (at 12 h), brain (at 6 h), and liver (at 48 h), but downregulated in the gill (at 24 h) by poly(I:C) stimulation (**Figure 6B**). Similarly, *Lm*Cx32.3 was induced in the head kidney, spleen after stimulation with poly(I:C), and LPS (**Figure 6C**). For *Lm*Cx32.7, LPS stimulation inhibited its expression in the head kidney and liver, whereas it was upregulated in the gill and liver after poly(I:C) stimulation (**Figure 6D**).

Like *Lm*Panx1, the expression of *Lm*Cx43 was upregulated to the different degrees, respectively, in the head kidney, gill, intestine, and liver after poly(I:C) or *E. tarda* stimulation (**Figure 6E**). In addition, *Lm*Cx43 was upregulated in all examined tissues except the gill after LPS stimulation, but was downregulated in the brain after *E. tarda* stimulation (**Figure 6E**).

### Expression Analysis of *Lm*Panx1, *Lm*Cx32, and *Lm*Cx43 after *In Vitro* Stimulation

**Figure 7:**
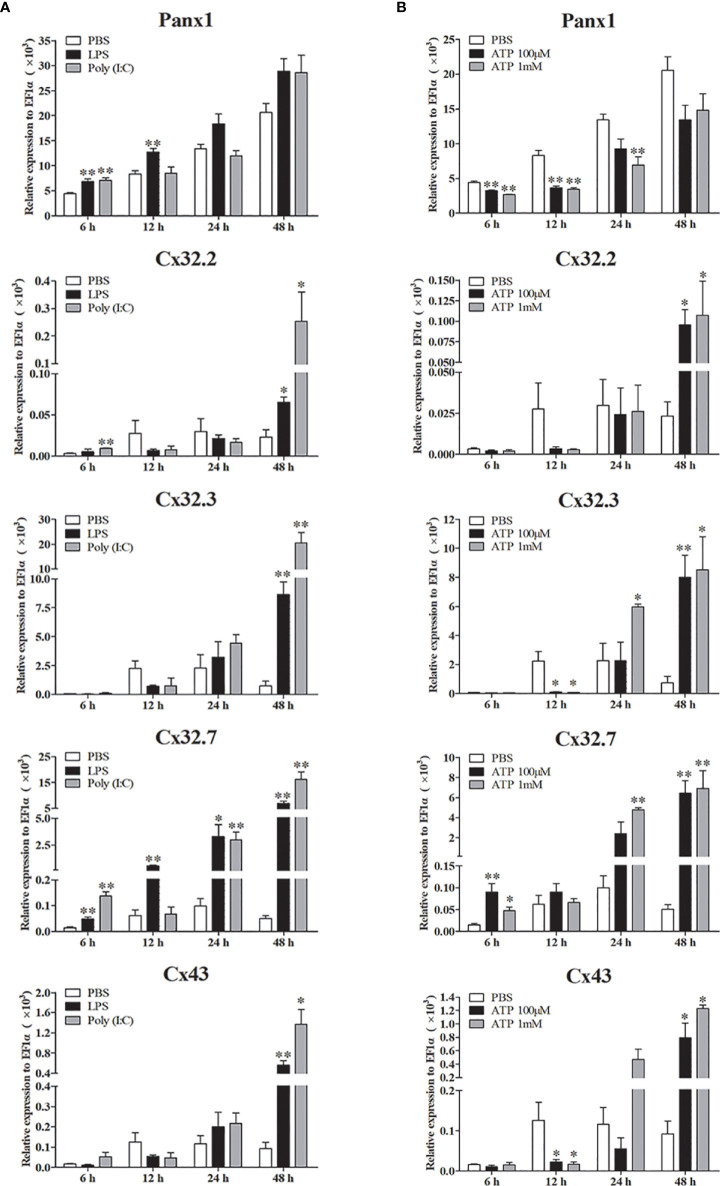
Expression of *Lm*Panx1, *Lm*Cx32, and *Lm*Cx43 in primary head kidney leukocytes after stimulation with LPS, poly(I:C) **(A)** or ATP **(B)**. Primary head kidney leukocytes were isolated from the spotted sea bass head kidney and stimulated with LPS (100 µg/mL), poly(I:C) (50 µg/mL), 100 µM ATP, 1 mM ATP, or PBS (control). The relative expression levels of target genes were normalized to that of EF1α. The data are shown as mean +SEM (N = 4). *p < 0.05, **p < 0.01 are considered significant difference.

Expression patterns of *Lm*Panx1, *Lm*Cx32, and *Lm*Cx43 were also analyzed in primary head kidney leukocytes after PAMP [LPS and poly(I:C)] and ATP stimulation (**Figure 7**). The leukocytes were treated with LPS (100 µg/mL), poly(I:C) (50 µg/mL), and ATP (100 µM or 1 mM), respectively. The cell samples were collected at 6, 12, 24, and 48 h after stimulation, and total RNA was then reversed to cDNA for qPCR. As shown in **Figure 7A**, all the genes were induced by PAMP stimulation. The *Lm*Panx1 expression was upregulated after LPS (at 6 h) and poly(I:C) (at 6 and 12 h) stimulation, whereas it remained unchanged at other stimulation conditions. Beyond that, all the genes except *Lm*Panx1 were upregulated at 48 h by PAMP stimulation. Among the three *Lm*Cx32 isoforms, *Lm*Cx32.7 responded most strongly to stimulation, and the expression of *Lm*Cx32.7 was obviously upregulated at all times except 12 h after poly(I:C) stimulation. According to the **Figure 7B**, the expression of three *Lm*Cx32 isoforms and *Lm*Cx43 was upregulated by ATP stimulation; nevertheless, when cells were stimulated with 100 µM or 1 mM ATP, the significantly downregulated expression of *Lm*Cx32.3 and *Lm*Cx43 was noted at 12 h. Conversely, treatment of 100 µM or 1 mM ATP inhibited the *Lm*Panx1 expression.

### Subcellular Localization of *Lm*Panx1, *Lm*Cx32, and *Lm*Cx43

**Figure 8:**
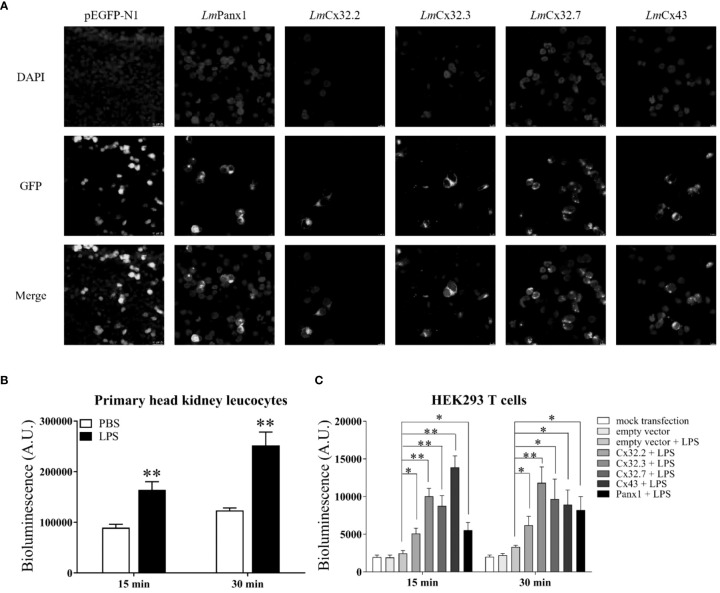
Subcellular localization of *Lm*Panx1, *Lm*Cx32, and *Lm*Cx43 in HEK293 T cells **(A)** and LPS-induced extracellular ATP release in primary head kidney leukocytes **(B)** or HEK293 T cells **(C)**. **(A)** HEK293 T cells were transfected with pEGFP-N1-*Lm*Panx1, pEGFP-N1-*Lm*Cx32.2, pEGFP-N1-*Lm*Cx32.3, pEGFP-N1-*Lm*Cx32.7, or pEGFP-N1-*Lm*Cx43 plasmids. At 24 h posttransfection, the cells were stained with DAPI and observed under a confocal microscope. **(B, C)** The primary head kidney leukocytes were stimulated with LPS (100 µg/mL) or PBS (control). HEK293 T cells were transfected with pcDNA3.1-*Lm*Panx1, pcDNA3.1-*Lm*Cx32.2, pcDNA3.1-*Lm*Cx32.3, pcDNA3.1-*Lm*Cx32.7, or pcDNA3.1-*Lm*Cx43. After 24 h, the cells were stimulated with LPS (100 µg/mL). The supernatant was collected at 15 and 30 min after stimulation and the ATP levels were subsequently measured. The mock transfected and empty plasmid transfected cells served as controls. Data are shown as mean +SEM (N = 4). *p < 0.05, **p < 0.01 are considered significant difference.

To determine the subcellular localization of *Lm*Panx1, *Lm*Cx32, and *Lm*Cx43, plasmids pEGFP-N1-*Lm*Panx1, pEGFP-N1-*Lm*Cx32.2, pEGFP-N1-*Lm*Cx32.3, pEGFP-N1-*Lm*Cx32.7, and pEGFP-N1-*Lm*Cx43 were constructed to express the GFP-tagged *Lm*Panx1, *Lm*Cx32.2, *Lm*Cx32.3, *Lm*Cx32.7, and *Lm*Cx43 fusion protein in the HEK293T cells, respectively. After 24-h culture, the cells were examined under a laser confocal microscope. In HEK293 T cells transfected with expression plasmids, GFP fluorescence was mainly located on the cell membrane, whereas in HEK293 T cells transfected with empty plasmids, GFP fluorescence was mainly located in the intracellular area (**Figure 8A**). The results showed that *Lm*Panx1, *Lm*Cx32, and *Lm*Cx43 were localized on the cellular membrane and can be expressed in HEK293 T cells.

### The Roles of *Lm*Panx1, *Lm*Cx32, and *Lm*Cx43 in LPS-induced Extracellular ATP Release

To investigate the function of *Lm*Panx1, *Lm*Cx32, and *Lm*Cx43 in inflammation-induced ATP release, primary head kidney leukocytes and HEK293 T cells (transfected with expression plasmids or empty plasmids) were stimulated with LPS, and the ATP levels were measured at 15 and 30 min after stimulation. In overexpression experiments, as negative controls, mock transfected and empty plasmid transfected cells were cultured in another 24-well plate at 24 h, but were not stimulated with LPS. As shown in **Figure 8B**, compared with the unstimulated, the levels of extracellular ATP have increased extremely significantly in primary head kidney leukocytes by LPS stimulation of 15 and 30 min. Furthermore, overexpression of *Lm*Panx1, *Lm*Cx32, or *Lm*Cx43 in HEK293 T cells, compared with cells transfected with empty plasmids, resulted in a significant increase in extracellular ATP levels after LPS stimulation at 15 or 30 min (**Figure 8C**).

## Discussion

Extracellular ATP has been shown to be an effective and conservative signaling molecule to activate natural immunity (8, 16). Therefore, it is important to study the molecular mechanisms of inflammation-induced ATP release. In mammals, substantial evidence indicates that Panx and Cx channels were participated in ATP release and had crucial immune functions (6). However, studies on the involvement of Panxs and Cxs in ATP release and natural immunity in fish remain limited. In the present article, Panx1, three Cx32 isoforms, and Cx43 were identified and characterized in *L. maculatus*, and these proteins share similar topological structure (**Figures 1** and **2**). Based on the results of our studies, Panx1 and Cx43 share similar genomic organization and synteny with their counterparts in vertebrates (**Figures 3** and **4**). In addition, there are multiple isoforms of Cx32 in selected fish, such as zebrafish (three isoforms), large yellow croaker (three isoforms), spotted gar (two isoforms), and rainbow trout (two isoforms). Accordingly, Cx32 gene containing duplicate copies may be a common characteristic in fish. It is worth noting that Cx32 and CX43 were located in the same locus in fish, but diverged into two loci from amphibian (**Figure 4B**), suggesting that during the evolution from fish to amphibians, Cx32 might have been transferred to other chromosomes.

Like their counterparts in mammals and other fish (such as Japanese flounder, zebrafish, and turbot), *Lm*Panx1, *Lm*Cx32, and *Lm*Cx43 were ubiquitously present in all tested tissues (**Figure 5**) (4, 21, 30, 32, 41). For instance, similar to Japanese flounder, the highest expression levels of *Lm*Cx32s (all of *Lm*Cx32.2, *Lm*Cx32.3, and *Lm*Cx32.7) and *Lm*Cx43 were found in the liver and brain, respectively. Interestingly, in mouse, Cx32 has been shown to be the major Cx protein in the liver, protecting the liver against liver injury (42), whereas Cx43 has been shown to be a “command gene” that regulates expression patterns, variability, and coordination of the brain transcriptome (43). Similar expression patterns suggest that *Lm*Cx32 and *Lm*Cx43 may play an active role in intercellular communications in tissues such as the liver and brain. Taken together, our results suggest that *Lm*Panx1, *Lm*Cx32, and *Lm*Cx43 may play distinct roles in different organs.

Studies have demonstrated that Panx1, Cx32, and Cx43 play important neural immune roles in mammals. For examples, Panx1 has been confirmed in inflammation of a variety of organs and tissues, especially the central and peripheral nervous system (44). Bacterial infection induced upregulation of Cx32 and Cx43 has also been demonstrated (45). In fish, Panx1, Cx32, and Cx43 were upregulated by PAMPs stimulation or bacterial infection (16, 31, 32). Similar to these studies, our findings showed that *Lm*Panx1, *Lm*Cx32, and *Lm*Cx43 were upregulated by PAMP [LPS and poly(I:C)] stimulation or *E. tarda* infection *in vivo* and *in vitro* (**Figures 6**, **7A**), suggesting the involvement of *Lm*Panx1, *Lm*Cx32, and *Lm*Cx43 in response to immune challenge in spotted sea bass. Interestingly, Panx1 and Cx43 are involved in mammalian neural inflammation, and multiple studies suggest that they could be targets for the treatment of neurological diseases in the future (6, 44). Therefore, we paid special attention to their expression after PAMP [LPS and poly(I:C)] and *E. tarda* infection in the brain. We found that *Lm*Panx1, *Lm*Cx32, and *Lm*Cx43 were downregulated in the brain at 6 or 12 h after stimulation, suggesting that the intercellular communication is hampered in the early stages of inflammation. Furthermore, we found that the three Cx32 isoforms and Cx43 were induced after ATP stimulation, but Panx1 was inhibited (**Figure 7B**). Interestingly, ATP may lead to hemichannel opening to release ATP by activating different purinergic receptors (46). Thus, our results suggest “ATP-induced ATP release” probably through the Cx hemichannels rather than the Panx1 hemichannel in fish.

Previous studies have demonstrated that among the Panx and Cx family proteins, Panx1, Cx32, and Cx43 are mainly expressed in several immune cells and participated in inflammation-induced ATP release in mammals. However, in fish, the functions of Cx32 and Cx43 in ATP release were found only in Japanese flounder (4, 21, 30). Moreover, bacterial and PAMP infection resulted in the release of ATP through Panx1 in tilapia and Japanese flounder (31). Thus, the evidence that Panx1 and Cxs were inflammation-induced ATP release in fish is still limited. In this study, we demonstrated that LPS can induce extracellular ATP release from primary head kidney leukocytes in spotted sea bass (**Figure 8B**). Second, we demonstrated that *Lm*Panx1, *Lm*Cx32, and *Lm*Cx43 were localized on the cellular membrane, which are necessary for ATP release from the channel (**Figure 8A**). Finally, we found that overexpression of *Lm*Panx1, *Lm*Cx32, or *Lm*Cx43 in HEK293 T cells leads to a significant increase in extracellular ATP levels (**Figure 8C**). Taken together, our results demonstrated that Panx1, Cx32, and Cx43 are participated in inflammation-induced ATP release in spotted sea bass.

In summary, Panx1, three Cx32 isoforms, and Cx43 were identified in spotted sea bass. Sequence analysis showed that these proteins share similar topological structure. Panx1 and Cx43 share similar genomic organization and synteny with their counterparts in selected vertebrates, but Cx32 is not very conserved. All the genes were upregulated by PAMP [LPS and poly(I:C)] stimulation or *E. tarda* infection *in vivo* and *in vitro*, but were downregulated in the brain at 6 or 12 h after stimulation. Furthermore, the three Cx32 isoforms and Cx43 were induced after ATP stimulation, but Panx1 was inhibited. More importantly, Panx1, Cx32, and Cx43 are involved in inflammation-induced ATP release in spotted sea bass. The results will contribute to further understanding of the innate immune response mediated by extracellular ATP in fish.

## Data Availability Statement

The datasets presented in this study can be found in online repositories. The names of the repository/repositories and accession number(s) can be found in the article/**Supplementary Material**.

## Ethics Statement

All fish experiments were conducted under the national regulations of laboratory animals of China and reviewed and approved by the ethics committee of laboratory animals of Shanghai Ocean University (SHOU-DW-2019-012).

## Author Contributions

ZS, YC, CX, and DL performed laboratory experiments and analyzed the data. QG and ZS designed the experiments. QG, PW, and ZS wrote the manuscript. All authors have read and approved the manuscript.

## Funding

This work was financially supported by the National Key Research and Development Program of China (no. 2018YFD0900605).

## Conflict of Interest

The authors declare that the research was conducted in the absence of any commercial or financial relationships that could be construed as a potential conflict of interest.

## Publisher’s Note

All claims expressed in this article are solely those of the authors and do not necessarily represent those of their affiliated organizations, or those of the publisher, the editors and the reviewers. Any product that may be evaluated in this article, or claim that may be made by its manufacturer, is not guaranteed or endorsed by the publisher.
