# Corrigendum: Characterization of pannexin1, connexin32, and connexin43 in spotted sea bass (*Lateolabrax maculatus*): they are important neuro-related immune response genes involved in inflammation-induced ATP release

**DOI:** 10.3389/fimmu.2024.1377603

**Published:** 2024-04-08

**Authors:** Zhaosheng Sun, Chong Xu, Yuxi Chen, Danjie Liu, Ping Wu, Qian Gao

**Affiliations:** ^1^ Key Laboratory of Exploration and Utilization of Aquatic Genetic Resources, Ministry of Education, Shanghai Ocean University, Shanghai, China; ^2^ International Research Center for Marine Biosciences at Shanghai Ocean University, Ministry of Science and Technology, Shanghai, China; ^3^ National Demonstration Center for Experimental Fisheries Science Education, Shanghai Ocean University, Shanghai, China; ^4^ College of Life Science and Technology, Huazhong University of Science and Technology, Wuhan, China

**Keywords:** pannexin1, connexin32, connexin43, innate immunity, ATP release, *Lateolabrax maculates*

## Error in Figure/Table

In the published article, there was an error in **Figure 8** as published. In **Figure 8A**, we mistakenly used a figure from our previous publication. The corrected **Figure 8** and its caption appear below.

**Figure 8:**
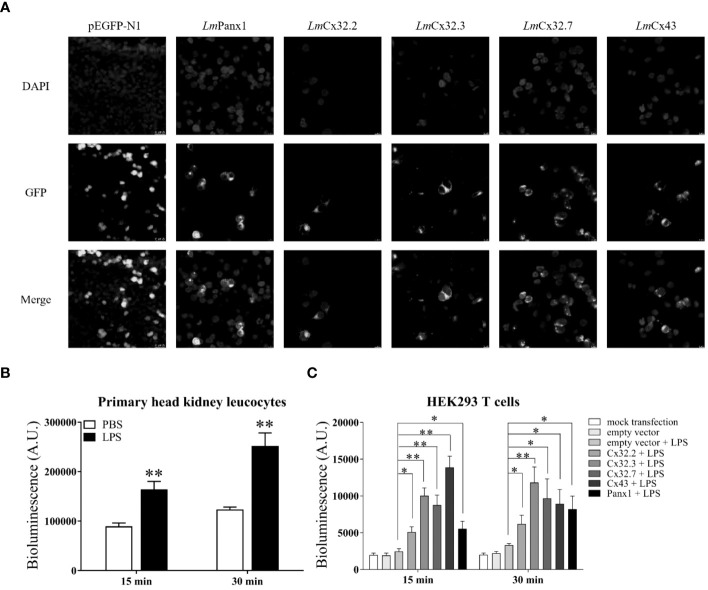
Subcellular localization of LmPanx1, LmCx32, and LmCx43 in HEK293 T cells **(A)** and LPS-induced extracellular ATP release in primary head kidney leukocytes **(B)** or HEK293 T cells **(C)**. **(A)** HEK293 T cells were transfected with pEGFP-N1-LmPanx1, pEGFP-N1 *Lm*Cx32.2, pEGFP-N1-*Lm*Cx32.3, pEGFP-N1-*Lm*Cx32.7, or pEGFP-N1-*Lm*Cx43 plasmids. At 24 h post transfection, the cells were stained with DAPI and observed under a confocal microscope. **(B, C)** The primary head kidney leukocytes were stimulated with LPS (100 µg/mL) or PBS (control). HEK293 T cells were transfected with pcDNA3.1-*Lm*Panx1, pcDNA3.1-*Lm*Cx32.2, pcDNA3.1-*Lm*Cx32.3, pcDNA3.1-*Lm*Cx32.7, or pcDNA3.1-*Lm*Cx43. After 24 h, the cells were stimulated with LPS (100 µg/mL). The supernatant was collected at 15 and 30 min after stimulation and the ATP levels were subsequently measured. The mock transfected and empty plasmid transfected cells served as controls. Data are shown as mean +SEM (N = 4). *p < 0.05, **p < 0.01 are considered significant difference.

The authors apologize for this error and state that this does not change the scientific conclusions of the article in any way. The original article has been updated.

