# Supplementary material for: Characterization of Pannexin1, Connexin32, and Connexin43 in Spotted Sea Bass (Lateolabrax maculatus): They Are Important Neuro-Related Immune Response Genes Involved in Inflammation-Induced ATP Release
Source: Front Immunol. 2022 Apr 19;13:870679. doi: 10.3389/fimmu.2022.870679 (PMC9062032; doi:10.3389/fimmu.2022.870679)
Supplement: Supplementary file 1 [file DataSheet_1.docx]

tcccatctgttgaatgttaggtctgcaacacgagctgaactccggtcctggaaatctcctac 62
aagtcacctgccgccatgaaactttaacagacagattacacttggcgggagcagtggttg 122
ttgttatttatgtgctgcaggtaacagaagagcggtgag**ATG**GCCATCGCCCACGTGGCT 182
  M  A  I  A  H  V  A  7
ACAGAGTACGTGTTCAGTGATTTTCTGCTGAAGGAGCCGAACCAGGCTCGATACCGCAAC 242
 T  E  Y  V  F  S  D  F  L  L  K  E  P  N  Q  A  R  Y  R  N  27
ATCCGAACAGAGCTGGCTGTGGACAAGATGGTGACCTGTGTGGCGGTGGGGCTTCCCCTC 302
 I  R  T  E  L  A  V  D  K  M  V  T  C  V  A  V  G  L  P  L  47
CTCCTCATCTCTCTGGCCTTCGCTCAAGAGGTTTCAGTCGGTGCTCAGATCAGCTGTTTT 362
 L  L  I  S  L  A  F  A  Q  E  V  S  V  G  A  Q  I  S  C  F  67
GCTCCTAGTAACTTCTCCTGGAAGCAGGCTGCTTATGTTGACTCGTACTGCTGGGCAGCT 422
 A  P  S  N  F  S  W  K  Q  A  A  Y  V  D  S  Y  C  W  A  A  87
ATACACACACACACTCTACCTCTGTGGCTGCACAAGTTCTTTCCTTATGTTTTGTTGCTG 482
 I  H  T  H  T  L  P  L  W  L  H  K  F  F  P  Y  V  L  L  L  107
GTGGCAGTGATGATGTACAGCCCGGCGTTGTTCTGGAGATTTTCTGCGGCGCCCCTCCTG 542
 V  A  V  M  M  Y  S  P  A  L  F  W  R  F  S  A  A  P  L  L  127
CAGTCGGACCTCAGCTTCATCATGGAGGAGTTGGACCGGTGTTACAACCGGGCCGTCACT 602
 Q  S  D  L  S  F  I  M  E  E  L  D  R  C  Y  N  R  A  V  T  147
CTGGCCAAACGCATGGCCACCTCAGGACAGCCCACCCCGGACAGCGACCCTACCGACGGC 662
 L  A  K  R  M  A  T  S  G  Q  P  T  P  D  S  D  P  T  D  G  167
TGTTTCAATTACCCGCTGTTGGAGAAGTTTCTGATGACCAAGCGCTGCTCGCGGGTGCTG 722
 C  F  N  Y  P  L  L  E  K  F  L  M  T  K  R  C  S  R  V  L  187
CTGTTTTACTACCTGCTGTGTCGCGGCCTGACTTTTGTCACCCTGCTGTGTGCCTGCATC 782
 L  F  Y  Y  L  L  C  R  G  L  T  F  V  T  L  L  C  A  C  I  207
TACCTGGGCTACTACCTCAAGCTGGCCTCCGTCACGGATGAGTTTGGCTGCCCGCTGCGT 842
 Y  L  G  Y  Y  L  K  L  A  S  V  T  D  E  F  G  C  P  L  R  227
GTTGGCCTGCCCGCCTTCGACCAGAGCGTCCCTGAAATGGTGCAATGTAAGCTCATTGCC 902
 V  G  L  P  A  F  D  Q  S  V  P  E  M  V  Q  C  K  L  I  A  247
GTGGGAGTCTTCTCTTTGCTGAGCCTCGTCAACTTGGTCCTGTTCATTGCACTAATTCCT 962
 V  G  V  F  S  L  L  S  L  V  N  L  V  L  F  I  A  L  I  P  267
GTGGTGATCTACGCCAGTCTCCGTCCCCTCTTCTGCCATGGCTACGCCAGCTTCCTGGAA 1022
 V  V  I  Y  A  S  L  R  P  L  F  C  H  G  Y  A  S  F  L  E  287
ACCTACCAATCACTGCCCACCGTCAGTGTCCTGCCCACCCCTGCTGGCCAATGGGACGAT 1082
 T  Y  Q  S  L  P  T  V  S  V  L  P  T  P  A  G  Q  W  D  D  307
CTCTCTCTGTATCTGCTCTTCCTGGAGGAGAACGTCAGCGAACTAAAGTCCTACAAATAT 1142
 L  S  L  Y  L  L  F  L  E  E  N  V  S  E  L  K  S  Y  K  Y  327
ATCAAGGTGTTGGAGTTGCTGAGGAGACGAGGTCATTGCTCCGGGGACAACTTCGACTCC 1202
 I  K  V  L  E  L  L  R  R  R  G  H  C  S  G  D  N  F  D  S  347
TTGGGTCTGCTGCAGGCTCTCTGTCTGGTGAAGATGGACAGCGTGGATGGGATTAAACCT 1262
 L  G  L  L  Q  A  L  C  L  V  K  M  D  S  V  D  G  I  K  P  367
ACCGGTGCTACAGGGAAACAGGATCAAGTAAAAACGAATGCCACGGACTCTGCCAGCAGC 1322
 T  G  A  T  G  K  Q  D  Q  V  K  T  N  A  T  D  S  A  S  S  387
CCCACAGCAGCTAAGAACAGCTGCAACCGTCCAAACTCAGCCGCCGATAACAGCAGCAAG 1382
 P  T  A  A  K  N  S  C  N  R  P  N  S  A  A  D  N  S  S  K  407
ATGGAAACTGAGATGAAAGAGCTCACTCCCTTGCTGCCAGAAAACAGCGACGTAACAGGC 1442
 M  E  T  E  M  K  E  L  T  P  L  L  P  E  N  S  D  V  T  G  427
AGCAATGAGAGAAAGACTCTCCGGCCGCAGGCGAAG**TGA**ccggctgctggcacagcaata 1502
 S  N  E  R  K  T  L  R  P  Q  A  K  -  439
gagacctggacaaaggggctgctgccactatctcatgggggcatctacaacttgtgtcct 1562
tcctgtcctgatgcagagatggaaatgggattgggagagtgtagcaccatcatgggtatc 1622
atccaagctccatcattcatcaccgtgggattcactgactctgatgcttttagtcctttt 1682
ctttgcatggtgtccttgtttggaagaaggaggagcagatttatttttattttttctggc 1742
atagcacaaagtgagaaagtgagtataaggtgatgtgagattagagtagacactggtaaa 1802
actagtaaaactcatgttggtacagatttcaggaatcattgattttattgaaaccattaa 1862
agactaatcccaatgtatctcttgagttaatggttaagacgctacggt**attaaa**atgctg 1922
acaaagatacaaaaaaaaaaaaaaaaaaaaaaaaaaa 1959

**Supplement Fig. 1. cDNA and deduced amino acid sequence of** *Lm*Panx1**.** The ORF is shown in upper case and the 5´-UTR and 3´-UTR sequences are in lower case. The translation initiation codon, stop codon and polyadenylation signal (attaaa) are shown in bold. The transmembrane domains (TM) are shaded in gray.

**A**

attcagccccacagcaggtgactggtctgtttgtctacacagagactctttgaattcaaaga 62
tcagacacc**ATG**GGAGAGTGGGGTTTTCTGTCCTCTCTCCTGGACAAGGTCCAGTCCCAC 122
     M  G  E  W  G  F  L  S  S  L  L  D  K  V  Q  S  H  17
TCCACCGTCATCGGGAAGGTCTGGCTCAGTGTGCTTTTCGTCTTCAGGATCATGATCCTT 182
 S  T  V  I  G  K  V  W  L  S  V  L  F  V  F  R  I  M  I  L  37
GGAGCTGGAGCAGAGAAGGTGTGGGGCGATGAACAGTCAAATATGATCTGTAACACCAAA 242
 G  A  G  A  E  K  V  W  G  D  E  Q  S  N  M  I  C  N  T  K  57
CAGCCTGGTTGCAAGAACGTCTGCTATGACCATGCCTTCCCAATCTCACACATTCGATTC 302
 Q  P  G  C  K  N  V  C  Y  D  H  A  F  P  I  S  H  I  R  F  77
TGGGTCCTCCAGATTATCTTTGTCTCAACACCGACTCTGATCTACCTTGGTCACGTCCTC 362
 W  V  L  Q  I  I  F  V  S  T  P  T  L  I  Y  L  G  H  V  L  97
CACATCATCCACAAAGAAAATAAAATCAGAGAATATATGAAGACTCACTCTCGGAGTGAA 422
 H  I  I  H  K  E  N  K  I  R  E  Y  M  K  T  H  S  R  S  E  117
ATCAACAAACTTCCCAAGTACTCTGACGAAAAAGGCCATGTTCAGATTAAAGGAGACCTG 482
 I  N  K  L  P  K  Y  S  D  E  K  G  H  V  Q  I  K  G  D  L  137
CTGGGAAACTACATGACCTCCATATTTTTCAGACTCATTCTGGAGGTAGCGTTCATTGTG 542
 L  G  N  Y  M  T  S  I  F  F  R  L  I  L  E  V  A  F  I  V  157
GGCCAGTATTATCTGTACGGGTTTGTCATGGACCCCAGAGTCGTCTGCTCCCGAGCCCCC 602
 G  Q  Y  Y  L  Y  G  F  V  M  D  P  R  V  V  C  S  R  A  P  177
TGTCCCTTCACTGTGGAGTGCTACATGTCTCGACCCACAGAGAAGACCATCTTCATCCTC 662
 C  P  F  T  V  E  C  Y  M  S  R  P  T  E  K  T  I  F  I  L  197
TTCATGCTTGTAGTGTCCTGTGTCTCTGTTCTCCTCAACGTAGTCGAGATCTTCTACCTG 722
 F  M  L  V  V  S  C  V  S  V  L  L  N  V  V  E  I  F  Y  L  217
GCGTGTTCTCGCTGCAGACGAAGGTCTAAAAGAGTGCAGTCTGCTTCTGTTGCCATTCAC 782
 A  C  S  R  C  R  R  R  S  K  R  V  Q  S  A  S  V  A  I  H  237
CCACGTTCAAACGGTGACAGTCTGATGAAAATTGAGAAGCTTGGCCTCCATGATGCCAGT 842
 P  R  S  N  G  D  S  L  M  K  I  E  K  L  G  L  H  D  A  S  257
CACAGCACAGCC**TAA**acaatgccatttagatccaatgtgaaaaatgtcacataggtacag 902
 H  S  T  A  -  261
aggaaacaggagggcaaataaaaatatttgagattgagattgatgaggctaaatccagaa 962
gagattatggcccacaggtgtaaattttcagagatatttatttatgtgatacttaaaaat 1022
tgctggaaaaaaaatgtgctttattttgtttctggctttgtattttgcacttacacctac 1082
atgatccagttttgagtattggcgcccccaagtggcaattggtaattgtaattggtttgt 1142
gactacatggactgactgtcagagggacctgaccatataacttgagtgcagatacctgtg 1202
tctctgtaccagtttacattggctaaaagccattttttagtgctgcaagctcttctttga 1262
aaagcagtgtgcaaggttagtcctactttacaaattgcaggcatgatgtttggactttca 1322
aatagtataattacaaatcatttgttttttcatgttttgtattttattgactgtatgtgc 1382
ctcatgtttataattgattgtaaaatatcataatgactgaggaaagggtacgcatatgtt 1442
t**attaaa**aaaatttcaagtgagcatttcaaaaaaaaaaaaaaaaaaaaaaaaaa 1496

**B**

atctctcccagccagagacagtcctttctgaccaggtcagcagatcagtaactgaacatca 61
ccgataaag**ATG**GGAGACTTTGGTTTTCTGTCAACCTTGCTGGACAAGGTCCAGTCCCAC 121
        M  G  D  F  G  F  L  S  T  L  L  D  K  V  Q  S  H  17
TCCACAGTCCTTGGGAAGATCTGGATGAGCGTCCTCTTCCTGTTCAGGATCATGGTTCTG 181
 S  T  V  L  G  K  I  W  M  S  V  L  F  L  F  R  I  M  V  L  37
GGTGCCGGTGCTGAGAGCGTCTGGGGGGACGAGCAGTCGGGTTTCATCTGCAACACTCAG 241
 G  A  G  A  E  S  V  W  G  D  E  Q  S  G  F  I  C  N  T  Q  57
CAACCTGGTTGTGAGAATGTCTGCTACGATTGGACCTTCCCCATCTCGCACATTCGCTTC 301
 Q  P  G  C  E  N  V  C  Y  D  W  T  F  P  I  S  H  I  R  F  77
TGGGTCCTCCAGATCATCTTCGTGTCCACGCCAACGCTGGTCTACCTGGGCCACGCCATG 361
 W  V  L  Q  I  I  F  V  S  T  P  T  L  V  Y  L  G  H  A  M  97
CACATCATCCACAAGGAGAACAAGATGAGGGAGCAGCTGTCGAGCCCAGGTGGGAAAAGG 421
 H  I  I  H  K  E  N  K  M  R  E  Q  L  S  S  P  G  G  K  R  117
CCCAAATACACAAACGAAAAGGGAAAGGTGACGATCAAGGGGAACCTGCTGGGGAGCTAC 481
 P  K  Y  T  N  E  K  G  K  V  T  I  K  G  N  L  L  G  S  Y  137
CTGACCCAGCTGGTGTTCAAGATCATCATTGAGGCTGCTTTCATTGTGGGCCAGTACTAC 541
 L  T  Q  L  V  F  K  I  I  I  E  A  A  F  I  V  G  Q  Y  Y  157
CTGTACGGCTTCATCATGGTCCCCATGTTCCCCTGCTCCAAGAAGCCCTGTCCCTTCACT 601
 L  Y  G  F  I  M  V  P  M  F  P  C  S  K  K  P  C  P  F  T  177
GTGGAGTGCTACATGTCCCGACCCACAGAAAAGACCATCTTTATCATCTTTATGCTGGTA 661
 V  E  C  Y  M  S  R  P  T  E  K  T  I  F  I  I  F  M  L  V  197
GTGGCCTGCATCTCCCTGCTTCTCAACTTCATTGAGGTGTTCTACCTGATTTGTACCAGG 721
 V  A  C  I  S  L  L  L  N  F  I  E  V  F  Y  L  I  C  T  R  217
GTCAGATGCGGGTCCAGGGCTCGCTCTCACAAGATCACGTCAGCAGAAAACCCTGCCAGC 781
 V  R  C  G  S  R  A  R  S  H  K  I  T  S  A  E  N  P  A  S  237
CTGTCGGCTCCCAGGTGGCCGACTGTAGAGGACGCACTCAAGCAGAACAAGATGAACATG 841
 L  S  A  P  R  W  P  T  V  E  D  A  L  K  Q  N  K  M  N  M  257
GAGCTGGAGAGTATTGGTGGAAGCCTGGATGGGGCCAAAGAGGAGAAACGACTGCTGAGT 901
 E  L  E  S  I  G  G  S  L  D  G  A  K  E  E  K  R  L  L  S  277
GGTCAT**TAA**aaaccgctgaccggaccattataatgtattaaatgctcttgttttagattt 961
 G  H  -  279
agaccacacagatccttgttcaggctgaagtagatagttcttacaatgttgtgtacaagt 1021
catgacagaacatcaagaaaaagctttatcattgtgtagcaaagtgatattattgtgaga 1081
aaagtctgcataaatttgccacgttgttggaattttacatttatgttgtattttggaaat 1141
ctcataatggaaagcgtgcaatattgataatgtatttatattcttcgatactcagtgaga 1201
gagcagaattttgtctatgcccagttattgaatgtgtcctttaagttattgccacattca 1261
tatgtagaatttgagataatttactcactgaatgatgatgatgatgatgatgatgatgtg 1321
tctttctactgtgtatggtagacatttttgtatgctgtattgttttt**aataaa**tgattca 1381
agttggataaaaaaaaaaaaaaaaaaaaaaaaaa 1415

**C**

aagcaggacaactggcgactgaa**ATG**GGTGAATGGGATCTTCTGGGCCGCCTGCTGGATAAA 62
      M  G  E  W  D  L  L  G  R  L  L  D  K  13
  GTGCAGAGCCACTCCACAGTGATCGGCAAGGTCTGGCTCACCGTGCTGTTTGTCTTCCGT 122
   V  Q  S  H  S  T  V  I  G  K  V  W  L  T  V  L  F  V  F  R  33
  ATCCTGGTCCTGCGCGCTGGCGCTGAGAAGGTGTGGGGCGATGAGCAATCTGACTTTGTC 182
   I  L  V  L  R  A  G  A  E  K  V  W  G  D  E  Q  S  D  F  V  53
  TGCAACACTCAGCAGCCCGGCTGTGAGAACGTCTGCTACGACTCCGCCTTCCCCATCTCT 242
   C  N  T  Q  Q  P  G  C  E  N  V  C  Y  D  S  A  F  P  I  S  73
  CACGTTCGCTTCTGGGTCCTTCAGATTATTGCTGTAGCGACTCCGAAGCTGTTATACCTT 302
   H  V  R  F  W  V  L  Q  I  I  A  V  A  T  P  K  L  L  Y  L  93
  GGACACGTCCTTCATGTGATCCACATTGAGAAGAAGGTGAAGGAGAAGATGAAGAAGCAG 362
   G  H  V  L  H  V  I  H  I  E  K  K  V  K  E  K  M  K  K  Q  113
  GCCGAGCTGGACGACCAGACCAGCCTGTTTCTTAGGAGGGCCTACAAAGTACCCAAGTAC 422
   A  E  L  D  D  Q  T  S  L  F  L  R  R  A  Y  K  V  P  K  Y  133
  ACCAAGAGCACGGGCAAGATCAGCATCCGTGGCCGTCTCCTTCGCAGTTATGTCTTCCAT 482
   T  K  S  T  G  K  I  S  I  R  G  R  L  L  R  S  Y  V  F  H  153
  CTTGTGGCCAAGATCGTCCTGGAGGTCTTGTTCATCGTGGGTCAGTACTTACTTTACGGT 542
   L  V  A  K  I  V  L  E  V  L  F  I  V  G  Q  Y  L  L  Y  G  173
  TTCACCCTCCAGACCCGCTACGTCTGCGACAGCTTCCCTTGCCCTCACAAGGTGGACTGC 602
   F  T  L  Q  T  R  Y  V  C  D  S  F  P  C  P  H  K  V  D  C  193
  TTCCTGTCCAGGCCTACGGAGAAGTCGGTCATCATCTGGTTCATGCTGGTGGCGGCGTCC 662
   F  L  S  R  P  T  E  K  S  V  I  I  W  F  M  L  V  A  A  S  213
  GTCTCCCTCGTCCTCAGTCTGGTTGAGCTGTTCTATCTGTGTGTGAAGGCTGTGAAGGAG 722
   V  S  L  V  L  S  L  V  E  L  F  Y  L  C  V  K  A  V  K  E  233
  TGCATGGCGAGGAGGCAGGACTACACCGTCACCCCGGTGACACCACCGCTTTCGGAAAGG 782
   C  M  A  R  R  Q  D  Y  T  V  T  P  V  T  P  P  L  S  E  R  253
  AAAGCTTTTAAAAGCCGCAGCGAGACGATCCAAAACTGTGTCAACCTGGAGCTGGAGCTC 842
   K  A  F  K  S  R  S  E  T  I  Q  N  C  V  N  L  E  L  E  L  273
  CAAGGACGAAAGTTAGGGGTGAACGGGGTCACAGGCGGCGTCAACGAGGTTGCCAAGAAC 902
   Q  G  R  K  L  G  V  N  G  V  T  G  G  V  N  E  V  A  K  N  293
  GTATCGCCTGAGAACAACAACATGGGGGAGGTCCACATC**TGA**agcatggaggccgtgata 962
   V  S  P  E  N  N  N  M  G  E  V  H  I  -  306
  ggtcgtacgtgtccagttctcctgagttccagtatttgtgtgctttggcgttggggaagt 1022

  taaacggtgtgtgtgtccagtaaataaagatgggggaagggaaagtagccagcttctgta 1082

  aacagaactccgtcacatgcaaaaatgttgttttaaagtgacataaaacaaaaataacac 1142

  tttaaattacttttttgatagattttgctaagggtaagtctttttgaatgatattagaca 1202

  actttgttacagttttgattcaataaacctcatattcagattcagattgtccttttcctg 1262

  ccatgttgtcatgtttgctcagtaaaccaacattttttgagatgc**aataaa**aagcaattt 1322

  ccctgcaaaaaaaaaaaaaaaaaaaaaaaaaaaaa 1357

**Supplement Fig. 2. cDNA and deduced amino acid sequence of** *Lm*Cx32.2 (A), *Lm*Cx32.3 (B) and *Lm*Cx32.7 (C)**.** The ORF is shown in upper case and the 5´-UTR and 3´-UTR sequences are in lower case. The translation initiation codon, stop codon and polyadenylation signal (attaaa or aataaa) are shown in bold. The transmembrane domains (TM) are shaded in gray.

aggtttgcctcccggtcccaaacttggatttcaacttcagcagagagagatctacacaat 60
tggcggagaaagtacctctgggccctctgttctttctctctgtgactttccaactaacgc 120
tccctgaga**ATG**GGTGACTGGAGTGCTCTGGGTCGTCTACTGGACAAGGTCCAGGCCTAC 180

        M  G  D  W  S  A  L  G  R  L  L  D  K  V  Q  A  Y  17
TCCACCGCTGGGGGGAAAGTGTGGTTGTCCGTCCTCTTCATATTCAGGATCCTGGTCCTC 240
 S  T  A  G  G  K  V  W  L  S  V  L  F  I  F  R  I  L  V  L  37
GGTACTGCGGTGGAATCAGCCTGGGGAGACGAGCAGTCTGCCTTCAAATGTAACACCCAG 300
 G  T  A  V  E  S  A  W  G  D  E  Q  S  A  F  K  C  N  T  Q  57
CAGCCTGGTTGTGAGAATGTCTGCTACGACAAATCCTTCCCCATCTCCCACGTTCGCTTC 360
 Q  P  G  C  E  N  V  C  Y  D  K  S  F  P  I  S  H  V  R  F  77
TGGGTCCTCCAGATCATCTTTGTGTCGACACCCACGCTCCTCTATCTGGCTCATGTCTTC 420
 W  V  L  Q  I  I  F  V  S  T  P  T  L  L  Y  L  A  H  V  F  97
TATCTAAACAGGAAGGAACAGAAATTCAACAGGAAGGAGGAGGAACTTAAAGCTGTGCAA 480
 Y  L  N  R  K  E  Q  K  F  N  R  K  E  E  E  L  K  A  V  Q  117
AATGATGGGGGTGATGTTGACATCCCGCTAAAGAAAATTGAGATGAAAAAGCTAAAGTAT 540
 N  D  G  G  D  V  D  I  P  L  K  K  I  E  M  K  K  L  K  Y  137
GGCATTGAGGAGCACGGAAAAGTGAAGATGAAGGGGGCCCTGCTCAGAACCTATATAGTC 600
 G  I  E  E  H  G  K  V  K  M  K  G  A  L  L  R  T  Y  I  V  157
AGCATTTTCTTCAAGTCTATGTTTGAGGTGGGCTTCCTGGTTATCCAGTGGTACATATAT 660
 S  I  F  F  K  S  M  F  E  V  G  F  L  V  I  Q  W  Y  I  Y  177
GGCTTCAGCCTGGCTGCAGTCTACACCTGTGAGAGGTCCCCATGTCCGCACAGAGTGGAC 720
 G  F  S  L  A  A  V  Y  T  C  E  R  S  P  C  P  H  R  V  D  197
TGTTTCCTGTCCCGACCCACAGAAAAGACAGTCTTCATCATCTTCATGTTGGTGGTGTCA 780
 C  F  L  S  R  P  T  E  K  T  V  F  I  I  F  M  L  V  V  S  217
CTGGTGTCCCTGCTGCTCAACGTCATTGAGCTTTTCTATGTGTTTTTTAAGAGGATCAAA 840
 L  V  S  L  L  L  N  V  I  E  L  F  Y  V  F  F  K  R  I  K  237
GATCGTGTGAAGGGCAAACAGCCGCCCACTCTCTACCCCAGTGCAGGCACCTTGAGCCCA 900
 D  R  V  K  G  K  Q  P  P  T  L  Y  P  S  A  G  T  L  S  P  257
ACCCCTAAAGATCTGTCCGCTGCTAAGTACGCTTATTATAACGGCTGTTCCTCCCCAACC 960
 T  P  K  D  L  S  A  A  K  Y  A  Y  Y  N  G  C  S  S  P  T  277
GCCCCACTCTCACCAATGTCCCCTCCAGGCTACAAGCTGGCCACAGGGGAGCGGGGAACT 1020
 A  P  L  S  P  M  S  P  P  G  Y  K  L  A  T  G  E  R  G  T  297
GGCTCATGCCGTAATTACAATAAGCAGGCCAATGAGCAGAACTGGGCCAACTACTCCACA 1080
 G  S  C  R  N  Y  N  K  Q  A  N  E  Q  N  W  A  N  Y  S  T  317
GAGCAGAACCGACTCGGCCAGAATGGTGGAGGAAGCACTATTTCAAACTCCCACGCACAA 1140
 E  Q  N  R  L  G  Q  N  G  G  G  S  T  I  S  N  S  H  A  Q  337
GCCTTTGACTTCCCCGATGATACCCACGAGCATAAGAAATTGTCCTCGTCAGCAGGACAA 1200
 A  F  D  F  P  D  D  T  H  E  H  K  K  L  S  S  S  A  G  Q  357
GAGCTGCAGCCACTGGCACTGATGGATGCTAGGCCCTGTAGCCGGGCCAGCAGCCGGATG 1260
 E  L  Q  P  L  A  L  M  D  A  R  P  C  S  R  A  S  S  R  M  377
AGTAGCCGGGCCAGGCCGGATGACCTGGATGTG**TAA**gcctcggctcctcctcccactcct 1320
 S  S  R  A  R  P  D  D  L  D  V  -  388
gcctggctgatgggatgtgtggcagcagccagggaatcaggaaacactcaatcacatagg 1380
ttcatatacgaggtggggctaacctcaatcactgttgtcaacatagagactcttagtcaa 1440
tctaagagacattaaaacttgttatgtaacactgtcaaaggtacaagttgcaaatatttt 1500
ttcaatgacttttaccataactttttagtagagctaactcagctttaagacaacagaaca 1560
gccacaggaatggtgcaagagtctatcagatgtactccatagatgaacagagggccagat 1620
ctaagtgactgatttggtttttatttgccacgcttacattgaagtaaaattctgttttcc 1680
tcttgttttcatagtttctgctacttttcctattggtgatagaaatgtacttagccctgt 1740
cataactgaagtctgaaggtatggtgacagctttacaacaaggggccttcttagttgtct 1800
aaaccagattaaaaatagcctttctaggctaactctggttaatttacagtcctttttttc 1860
tgtttatcaatgaacaacctgtcaaataaacaaatacataaaaggagagtcaagtaggga 1920
aagaaacaaatttccaaacccacgtgtcaaatccctaatttagccaaatggtctaaatca 1980
tataatttggaactgagaaagtaaaggtgaacacatccagaatgtccattcggactgcag 2040
ggaatcattctaactgcaaccgtggacagatgacatatatgacagctatggaagctttct 2100
gatctgccactcctgtggctaaggtaaataaaactatgtagttaaggttagggaaaggtc 2160
tcctttatggttataaaagcaacactgagttttggtaggagctaggatgtaaactctcca 2220
gcgttgacgttacttgctttctttatccctgagagatattactgtagaaagatatctcac 2280
ttcttccacctcagctcctgagagaggacagtcatcgcaagtaccacctcaaaagccaaa 2340
acaggtaaacatagttcatatcagcgatctgacaaatgctattgtttttcaggtgaggac 2400
agtcgcttgctttgttataaaactgcttgatgcatctgtgcactttgttataaagctgtg 2460
tgtagtccatccatccatttattatctataccgcttatccttagagggtcgcgtgggggg 2520
gctggagccaatcctagctgtgtgtagttcaagaaactggca**aataag**ttaatatcacaa 2580
ccaacaggaatgatgaaaaaaaaaaaaaaaaaaaaaaaaaaaaaa 2625

**Supplement Fig. 3. cDNA and deduced amino acid sequence of** *Lm*Cx43**.** The ORF is shown in upper case and the 5´-UTR and 3´-UTR sequences are in lower case. The translation initiation codon, stop codon and polyadenylation signal (aataag) are shown in bold. The transmembrane domains (TM) are shaded in gray.
